# Distribution of pulmonary ventilation in women with post-COVID-19 before and after the use of a respiratory incentive device (UBICU): a pilot study

**DOI:** 10.3389/fdgth.2026.1789878

**Published:** 2026-04-13

**Authors:** Esther Cecilia Wilches-Luna, Valeria Perez-Hortúa, Helberg Asencio-Santofimio, Jaime Aguilar-Zambrano, Leonardo Arzayus-Patiño

**Affiliations:** 1 Human Rehabilitation School, Universidad del Valle, Cali, Colombia; 2 Department of Health Basic Sciences, Pontificia Universidad Javeriana Cali, Cali, Colombia; 3 Department of Electronics and Computer Science, Pontificia Universidad Javeriana Cali, Cali, Colombia

**Keywords:** COVID-19, electrical impedance tomography, incentive spirometry, pulmonary rehabilitation, respiratory function tests

## Abstract

**Introduction:**

In the aftermath of the COVID-19 pandemic, restrictive pulmonary complications have emerged as a common long-term sequela. To address these impairments, a novel flow-based respiratory incentive device, UBICU, was developed to promote lung expansion through gamification and visual feedback. The aim of this study was to describe the pulmonary ventilation distribution using Electrical Impedance Tomography (EIT) in women with long COVID and restrictive pulmonary impairment, before and after using the UBICU device.

**Methods:**

This exploratory (pre-post) pilot study included eight women with post–COVID-19 restrictive impairment, as determined by spirometry in accordance with ATS/ERS guidelines. Pulmonary ventilation distribution was assessed before and after the intervention using electrical impedance tomography. Participants were provided with the UBICU device and instructed to perform three sets of 10 repetitions daily for seven consecutive days. The study was approved by the Institutional Ethics Committee, adhered to the principles of the Declaration of Helsinki, and written informed consent was obtained from all participants.

**Results:**

An asymmetric regional ventilation pattern was observed before and after the intervention. Statistically significant improvements were found in ROI 1 (*p* = 0.007), ROI 3 (*p* = 0.007), and ROI 4 (*p* = 0.007) after using the UBICU device.

**Conclusion:**

Use of the UBICU device was associated with improvements in pulmonary ventilation distribution, suggesting its potential as an adjunctive therapeutic tool in the management of women with long COVID and restrictive pulmonary impairment.

## 1 Introduction

The World Health Organization (WHO) defines post-COVID-19 condition, or long COVID, as the persistence or emergence of new symptoms three months after the initial SARS-CoV-2 infection, lasting for at least two months and not attributable to another cause. This condition can affect any individual exposed to the virus, regardless of age or severity of the acute phase (1).

In patients with long COVID, various pathophysiological mechanisms such as endothelial dysfunction, microthrombosis, inflammation, and pulmonary fibrosis converge to significantly alter the distribution of ventilation. This confluence of factors leads to ventilation/perfusion (V/Q) mismatch, resulting in a heterogeneous pattern of pulmonary ventilation (2).

Although the proportion of symptoms directly related to pulmonary sequelae remains unclear, the most frequently reported persistent respiratory symptom in the literature is dyspnea (3), followed by cough and chest pain. Pulmonary fibrosis secondary to acute lung injury has also been observed, and is associated with the severity of the initial infection, intensive care unit (ICU) admission, and prolonged immobilization. Some authors have identified that approximately one-third of COVID-19 survivors present with fibrotic changes that impair ventilation (4).

Scientific evidence supports the high incidence of post-COVID-19 sequelae, establishing it as a public health concern due to the level of disability it causes (5). This condition significantly affects quality of life and hinders patients' return to work, social integration, and family life (6, 7). The prevalence of residual symptoms after the acute phase ranges from 43% to 62%, with fatigue and dyspnea being the most prominent, regardless of the initial severity of the disease, according to a meta-analysis of 194 studies (8).

Long COVID is associated with risk factors such as advanced age, cardiovascular diseases, and diabetes, with female sex identified as a significant predisposing factor (9). A multicenter cohort study revealed that, although initial COVID-19 symptoms in hospitalized patients were similar between men and women, the latter were more likely to develop post-COVID-19 symptoms eight months after hospital discharge (10). Bai et al. reported that women are up to three times more likely to be diagnosed with this condition, regardless of age (11).

Several studies have reported that women exhibit a higher predisposition to developing long COVID, presenting with persistent respiratory symptoms such as dyspnea and fatigue. This suggests alterations in pulmonary function and, consequently, ventilation distribution (10–12). However, there is a notable lack of research specifically examining sex-related differences in ventilatory abnormalities associated with long COVID-19, particularly using physiological imaging techniques.

Electrical Impedance Tomography (EIT) emerges as a valuable tool for assessing pulmonary function. It enables real-time monitoring of regional ventilation distribution, facilitating the accurate detection of areas with abnormalities and the objective quantification of ventilation heterogeneity. Its utility has been demonstrated in the context of acute COVID-19 (13–16), particularly in ICU settings, guiding alveolar recruitment maneuvers and optimizing mechanical ventilation due to its ability to provide bedside, radiation-free, real-time data. However, its application as an outcome measure to evaluate regional ventilation changes in patients with long COVID undergoing respiratory interventions remains limited. Given the persistence of respiratory symptoms in long COVID-19, EIT presents a promising option for evaluating ventilation in ambulatory care settings.

International guidelines recommend ongoing intervention and monitoring of respiratory impairment in patients with long COVID-19. Scoping reviews suggest that respiratory rehabilitation—from hospital admission through outpatient follow-up,benefits these patients by improving lung function (8), and some studies have proposed the use of incentive spirometry (IS) as a strategy to support this recovery. Nevertheless, most available evidence focuses on global functional outcomes rather than on regional ventilatory mechanisms.

These characteristics distinguish UBICU from conventional incentive spirometers evaluated in previous post-COVID-19 studies. Within this context, we developed UBICU, a novel IS device (Utility Model Patent No. 42,109, Superintendence of Industry and Commerce of Colombia) aimed at improving adherence to respiratory physiotherapy. Unlike conventional IS devices (17), UBICU is a flow-based system that integrates dedicated hardware with companion software to improve usability during exercise performance and to generate quantitative metrics for physiotherapists to assess execution quality. The platform also enables remote prescription and monitoring of multiple patients through a cloud-based administration interface. UBICU provides real-time visual feedback via an Android application running on a smartphone or tablet connected wirelessly through Bluetooth and incorporates gamification strategies to enhance engagement in lung re-expansion exercises. Gamification has been reported as a beneficial approach in therapeutic settings, as it may reduce anxiety, shift attention away from discomfort during task execution, and support sustained participation in treatment (18). Exercise prescriptions and performance data are transmitted to the cloud in real time via Wi-Fi, enabling continuous monitoring and timely clinical decision-making.

Chen et al. (19) published a narrative review on the impact of IS use in long COVID-19. This review highlighted the limited existing evidence on IS for symptom improvement in long COVID-19, emphasizing the urgent need for further research to assess its long-term utility, particularly in outpatient settings. **I**mportantly, none of the studies included in this review incorporated physiological imaging techniques such as EIT to assess regional ventilation changes.

The limited research in Colombia on the pulmonary impact of long COVID in women represents a critical knowledge gap with direct implications for public health and decision-making. There is an urgent need for studies characterizing pulmonary involvement in this population, including assessments of lung function and ventilation distribution, to support therapeutic interventions such as incentive spirometry. Accordingly, this exploratory study was designed not only to assess global pulmonary function but also to characterize regional ventilation patterns using EIT**.** The primary objective of this exploratory study was to describe pulmonary ventilation distribution using electrical impedance tomography (EIT) in adult women with long COVID and restrictive pulmonary impairment, before and after using the UBICU device.

## 2 Materials and methods

### 2.1 Study design

A descriptive, exploratory (pre-post) pilot study was conducted between May and August 2023. All participants provided informed consent after fully understanding the study procedures. The protocol was approved by the Institutional Research Ethics Committee (Approval No. 012-2021) and was carried out in accordance with the guidelines established in Resolution 8,430 of 1,993 of the Colombian Ministry of Health and the ethical principles of the Declaration of Helsinki.

### 2.2 Population

The target population consisted of individuals previously diagnosed with COVID-19 through reverse transcription polymerase chain reaction (RT-PCR) and presenting with restrictive pulmonary impairment as defined by the American Thoracic Society (ATS) (20).

Inclusion criteria were: adult volunteers over 18 years old with a prior confirmed diagnosis of COVID-19, presenting with restrictive lung impairment defined by a Forced Vital Capacity (FVC) less than 80% of predicted, no cognitive or mental impairments, and access to internet connectivity. Exclusion criteria included: pregnant women, individuals with obstructive pulmonary impairment, tracheostomy, implanted pacemakers, poor tomographic signal quality, and those unable to comprehend or use the UBICU application for the breathing sessions.

### 2.3 Sample

A non-probabilistic convenience sampling method was used to recruit eight women. This methodological decision was based on the logistical challenges of accessing a specific clinical population with a confirmed diagnosis of long COVID and restrictive ventilatory impairment in an outpatient setting and within a limited time frame. Given the exploratory and preliminary nature of the study, this approach allowed the collection of initial data to characterize the ventilatory response to the UBICU device in a population underrepresented in the scientific literature. Although this methodology is acknowledged to limit the external validity and generalizability of the results, the findings should be interpreted as an initial approximation intended to lay the groundwork for future research with broader scope and greater methodological rigor.

### 2.4 Instruments

A data collection form was designed to record sociodemographic variables (gender, age, socioeconomic status, marital status, and occupation); anthropometric data [weight, height, and Body Mass Index (BMI)]; clinical variables (personal medical history, hospitalization, and supplemental oxygen requirement); and physiological variables (respiratory rate, heart rate, and oxygen saturation).

Pulmonary function was assessed using the Spirobank II spirometer, with the following variables recorded: Forced Expiratory Volume in one second (FEV₁), Forced Vital Capacity (FVC), and the FEV₁/FVC ratio. Tomographic variables were obtained using the Dräger PulmoVista 500 electrical impedance tomograph. Measurements included global and regional tidal variation per minute (TVM), representing the difference between the minimum and maximum values of the impedance curve for each breath within one minute, and the percentage distribution of pulmonary ventilation across four regions of interest (ROIs), divided into quadrants for this study.

The UBICU flow-oriented incentive spirometer, based on integrated hardware and software technology, was used. UBICU quantifies inspiratory airflow using a differential pressure method through a Venturi system with integrated pressure transducers, enabling precise measurement of inspiratory flow instead of the qualitative visual shift typical of conventional incentive spirometers. This quantitative approach forms the technological basis of the device and supports its clinical use (21).

UBICU also includes an interactive digital platform that provides real-time visual feedback, therapeutic gamification, and a reward system to improve patient engagement and adherence. The interface informs patients about the achievement of inspiratory goals and progressively reinforces training effort.

The device allows for near real-time remote monitoring, as session data is transmitted via a mobile application to a cloud platform accessible to physiotherapists. This interface allows for the remote adjustment of training targets within the game environment, facilitating the modulation of inspiratory demand based on patient performance and enabling individualized prescription (21).

Table 1 presents a structured comparison between UBICU and conventional incentive spirometers, outlining the differences in measurement principles, feedback, remote monitoring, and therapeutic adaptability. Prior to clinical implementation, UBICU was evaluated in healthy individuals to verify its technical performance and measurement reliability compared to commercially available devices. This preliminary validation confirmed its feasibility, and the present study represents the first clinical application of UBICU in a patient population.

**Table 1:** Comparison between conventional incentive spirometers and the UBICU device, highlighting differences in flow measurement principles, feedback mechanisms, remote monitoring, and therapeutic adaptability.

Feature	Conventional incentive spirometer	UBICU device
Operating principle	Visual displacement or estimated flow-based mechanism	Quantitative measurement of inspired flow based on pressure differential (Venturi system)
Measurement accuracy	Qualitative or semi-quantitative	Quantitative, based on pressure transducers
Technological protection	Not applicable	Utility model patent
Patient feedback	Simple, passive visual feedback	Interactive real-time visual feedback
Gamification	No	Yes (therapeutic game-based system)
Reward system	No	Yes, linked to completion of prescribed sessions
Adjustment of therapeutic targets	Fixed, not dynamically adjustable	Remotely adjustable by the physiotherapist via the application, indirectly modifying inspiratory demand
Performance monitoring	Not available	Remote monitoring in near real time
Data transmission	Not available	Data transmitted to a cloud-based platform via a mobile application
Remote clinical assessment	Not possible	Allows remote evaluation and follow-up by the physiotherapist
Session recording	Not available	Automatic recording of each training session
Suitability for outpatient rehabilitation	Limited	High, particularly in post-COVID-19 scenarios
Integration with physiological imaging techniques	Not reported	Evaluated in combination with electrical impedance tomography (EIT) in the present study

### 2.5 Evaluation

Interested participants were scheduled for an initial session, during which the study was explained, and informed consent was obtained. Spirometry was then conducted following ATS/ERS guidelines regarding measurement technique, equipment calibration, and acceptability and reproducibility criteria. Individuals with FVC less than 80% of predicted and below the lower limit of normal for their height and weight were invited for tomographic measurements 24 h later. Participants were instructed to abstain from physical activity and smoking for 12 h prior.

**Figure 1:**
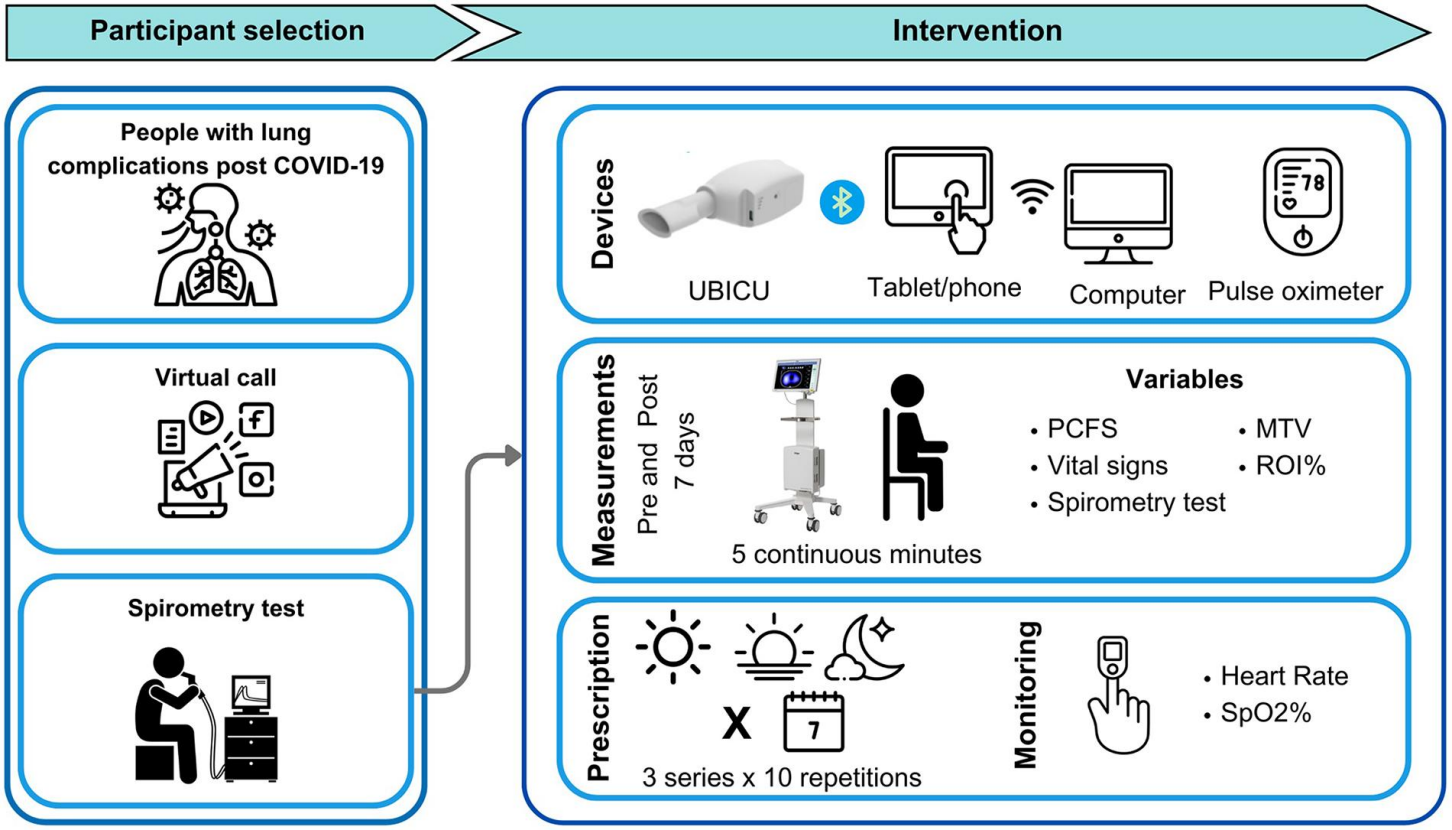
Measurement and intervention process of the participants.

To assess functional status, the post-COVID-19 Functional Status (PCFS) Scale, translated, validated, and adapted for the Colombian population, was used. This scale classifies individuals into five levels: no functional limitations, negligible, slight, moderate, and severe functional limitations (22) (Figure 1).

### 2.6 Measurements

#### 2.6.1 Anthropometric measurements

Anthropometric data (height and weight) and sociodemographic and clinical variables were recorded prior to tomographic assessment.

#### 2.6.2 Tomographic measurements

Pulmonary ventilation distribution was evaluated using EIT both before and after seven days of UBICU use. The PulmoVista 500 injects an alternating current through a pair of electrodes, with voltage amplitudes recorded by adjacent electrodes to create an impedance profile. A total of 208 values were generated and processed through a modified Newton-Raphson algorithm to reconstruct a real-time dynamic cross-sectional image. Each EIT image consisted of a 32 × 32-pixel matrix (23).

All measurements were performed after automatic self-check and calibration of the equipment. Each participant was connected to the PulmoVista 500 via a belt with 16 electrodes placed around the thoracic wall between the fifth and sixth intercostal spaces, according to manufacturer recommendations. Prior to starting the tomographic measurement, participants were seated in a chair with back support and instructed to breathe normally at tidal volume without speaking. A five-minute recording of quiet breathing was obtained. Figure 1 describes the evaluation and measurement process.

After the recording and removal of the belt, a different researcher (from the one conducting the tomographic measurements) provided the participant with video-based instructions on how to properly use the UBICU device.

#### 2.6.3 Instructions for using the app and UBICU

An engineer provided a mobile device or tablet with the UBICU app and explained the connection process. An instructional video was provided to demonstrate proper device use.

#### 2.6.4 Exercise prescription

A physiotherapist created a user profile in the app, entering weight, age, and sex, and prescribed three sets of 10 repetitions every 8 h, with one-minute intervals between sets. The physiotherapist and engineer maintained remote communication with the participant to address questions and ensure continued monitoring.

#### 2.6.5 Monitoring

Participants were instructed to record oxygen saturation and heart rate before, during, and after each UBICU session.

#### 2.6.6 Remote follow-Up

**Figure 2:**
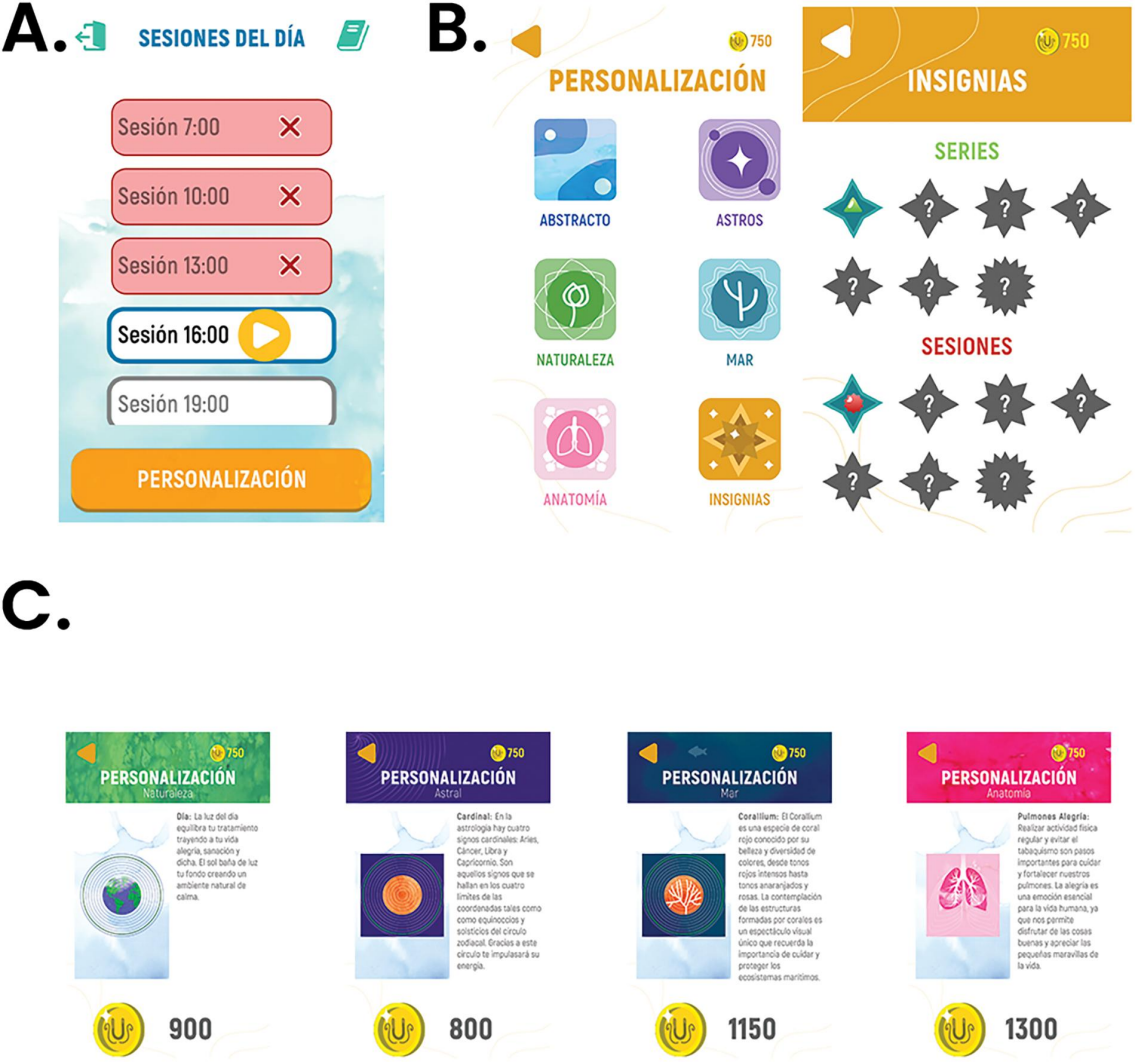
Screenshot of the UBICU mobile application. The patient-specific prescribed exercise schedule **(A)** UBICU gamification and personalization features **(B)** Examples of user-selectable personalization options **(C)** *The images are in Spanish because it is the original language of the application.*.

To ensure correct exercise execution, we maintained regular communication with participants to address concerns and provide technical support. The UBICU application was configured to present the prescribed exercise schedule (Figure 2A) and to guide patients through animated, interactive feedback. The gamification component provided ongoing reinforcement by awarding “UBICoins” based on patient effort; these virtual rewards could be used to unlock additional scenarios and obtain in-app recognition (Figure 2).

## 3 Statistical analysis

The EIT sessions were stored on a USB drive, labeled with the assigned participant code, and transferred to a computer for the reconstruction of EIT measurements using the Electrical Impedance Tomography Data Analysis Tool v6.3 (Draeger Medical). A researcher who did not participate in the measurements conducted random reviews and quality control checks to verify the accuracy of the information before entering it into the database.

Data were entered into Microsoft Excel 2020 and then exported to SPSS software for analysis. Descriptive statistics were performed.

The Shapiro–Wilk test was applied to assess the normality of quantitative variables. Variables with a normal distribution are expressed as mean and standard deviation; those with a non-normal distribution are presented as median and interquartile range. Qualitative variables are reported as frequencies and percentages.

To compare pulmonary function and ventilation distribution before and after the use of the UBICU device, paired comparisons were initially performed using the paired Student's *t*-test. Given the small sample size (*n* = 8) and the exploratory nature of the study, normality of paired differences was assessed using the Shapiro–Wilk test.

Additionally, to reinforce the robustness of the results, non-parametric analyses using the Wilcoxon signed-rank test were conducted as sensitivity analyses for all pre–post comparisons. A significance level (alpha) of 5% was established for statistical inference. Results were considered consistent when both parametric and non-parametric approaches led to the same conclusions.

## 4 Results

From June to August 2023, eight individuals who met the inclusion criteria for the study were recruited (Figure 2). The average age of the participants was 63 ± 9 years, and all participants were women. According to the IPAQ, 75% of the participants had a low level of physical activity, and 50% had a Charlson morbidity index of 20% (Table 2).

**Table 2:** Characterization of the participants.

Variables	Total *n* = 8	
Sex, female (%)	100	
Age^a^	63 ± 9	
Educational level		
Technical	2 (25%)	
University	4 (50%)	
Postgraduate	2 (25%)	
Anthropometric measurements^a^		
Height (m)	1.55 ± 9.5	
Weight (kg)	61 ± 15.5	
BMI (kg/m^2)^	25 ± 4	
Comorbidities		
High blood pressure	5 (62.5%)	
Diabetes	2 (25%)	
Hypothyroidism	1 (12.5%)	
Hospitalization for COVID-19		
Yes	5 (62.5%)	
Supplementary oxygen requirement		
Yes	5 (62.5%)	
Level of physical activity IPAQ		
Low	6 (75%)	
Medium	2 (25%)	
High	0 (0%)	
Charlson index		
22% mortality	4 (50%)	
50% mortality	3 (37.5%)	
70% mortality	1 (12.5%)	
Vital signs during EIT assessment^a^		
	*Before*	*After*
Respiratory rate (breaths/min)	14 ± 1,6	15 ± 1,3
Heart rate (bpm)	66 ± 21.8	78 ± 9.5
Oxygen saturation (%)	95 ± 1.5	95 ± 0.8
Final score of the evaluator PCFS scale		
	*Before*	*After*
No functional limitations	*2* (*25%)*	*4 (50%)*
Negligible functional limitations	*4* (*50%)*	*3 (37.5%)*
Slight functional limitations	*2* (*25%)*	*1 (12.5%)*
Lung function characteristics^a^		
	*Before*	*After*
FVC	71 ± 1.7	76 ± 0.8
FEV1	83.2 ± 7.4	87 ± 8.2
FVC/FEV1	92 ± 4	94 ± 15.6**

N, sample; BMI, body mass index; FVC, forced vital capacity; FEV1, forced expired volume in 1 s; FEV1/FVC, fraction of air that an individual exhaled in one second with respect to forced vital capacity; m, meters;.

PCFS, Post-COVID-19 Functional Status; EIT, electrical impedance tomography bpm; beats per minute; breaths/min, breaths per minute.

^a^ values expressed as the mean and standard deviation.

** *p* < 0.05.

### 4.1 Global Minute Tidal Variation

**Figure 3:**
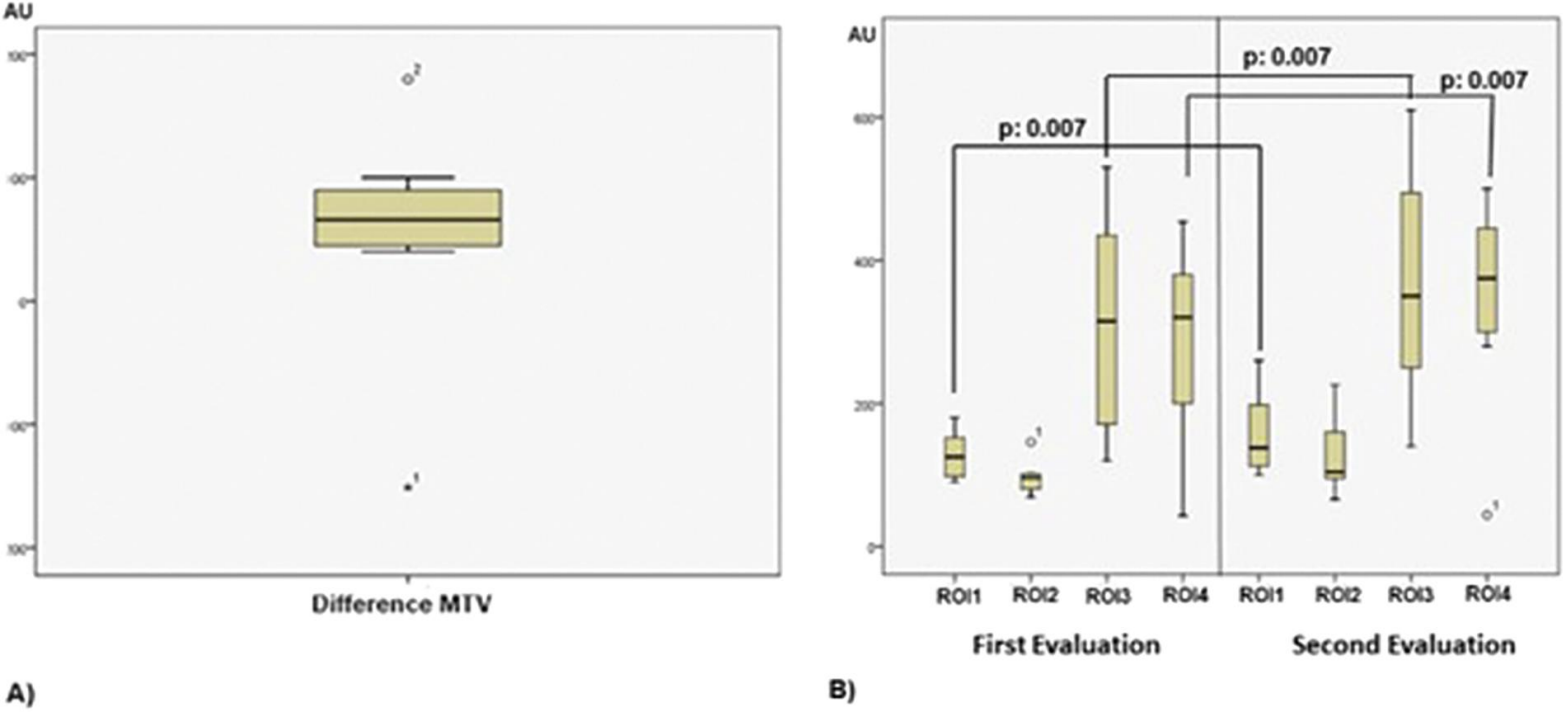
GLOBAL and regional MTV: before vs. after. **(A)** Global MTV difference. **(B)** Regional MTV first and second evaluation.

An increase in global Minute Tidal Variation (MTV) of 53.8 arbitrary impedance units (AUs) (95% CI: 132.0 to −24.3) was observed after 7 days of using the UBICU spirometer. However, this change was not statistically significant (*p* = 0.14) (Figure 3A). This finding remained consistent when analyzed using the non-parametric Wilcoxon signed-rank test.

### 4.2 Regional Minute Tidal Variation

**Figure 4:**
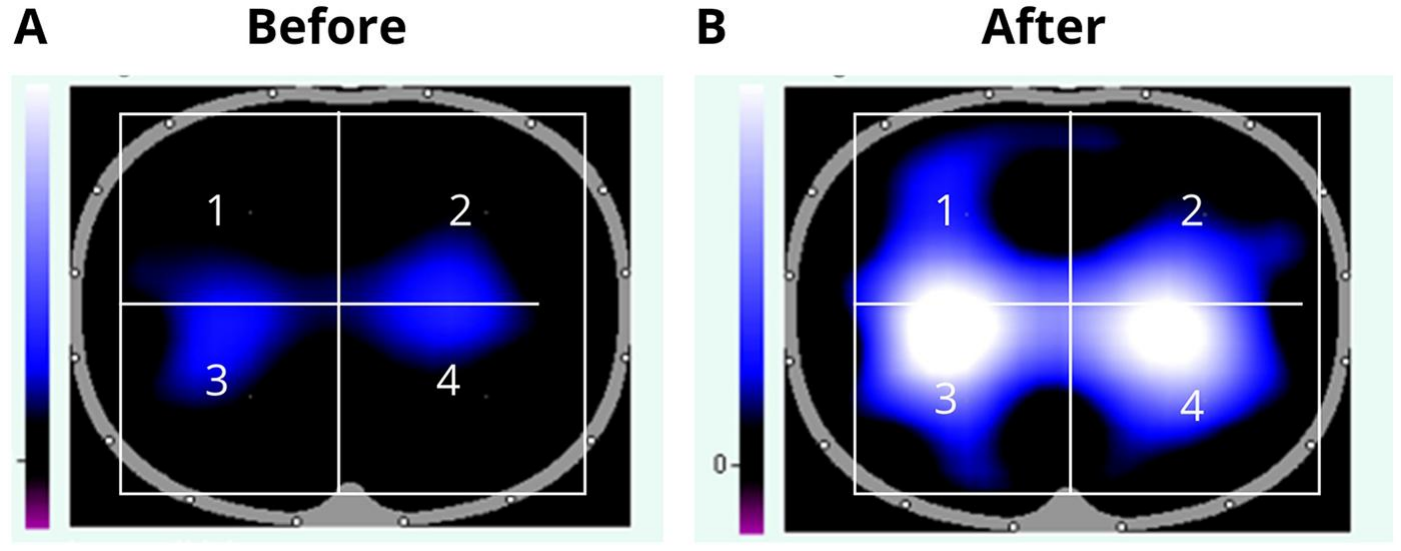
Distribution of regional pulmonary ventilation before **(A)** the use of the UBICU spirometer and 7 days after **(B)** the following figure shows the comparison of pre- and post-UBICU use in one of the participants.

The distribution of regional MTV differed before and after the use of the UBICU spirometer. There was a significant increase in the percentage of ventilation distribution in ROI 1 (mean difference = 29.63, 95% CI: 0.51 to 58.74, *p* = 0.007), ROI 3 (mean difference = 55.88, 95% CI: 24.58 to 87.17, *p* = 0.007), and ROI 4 (mean difference = 48.14, 95% CI: 11.55 to 84.74, *p* = 0.007) (Figures 3B, 4). These results remained statistically significant when confirmed using the Wilcoxon signed-rank test.

### 4.3 Pulmonary function

**Figure 5:**
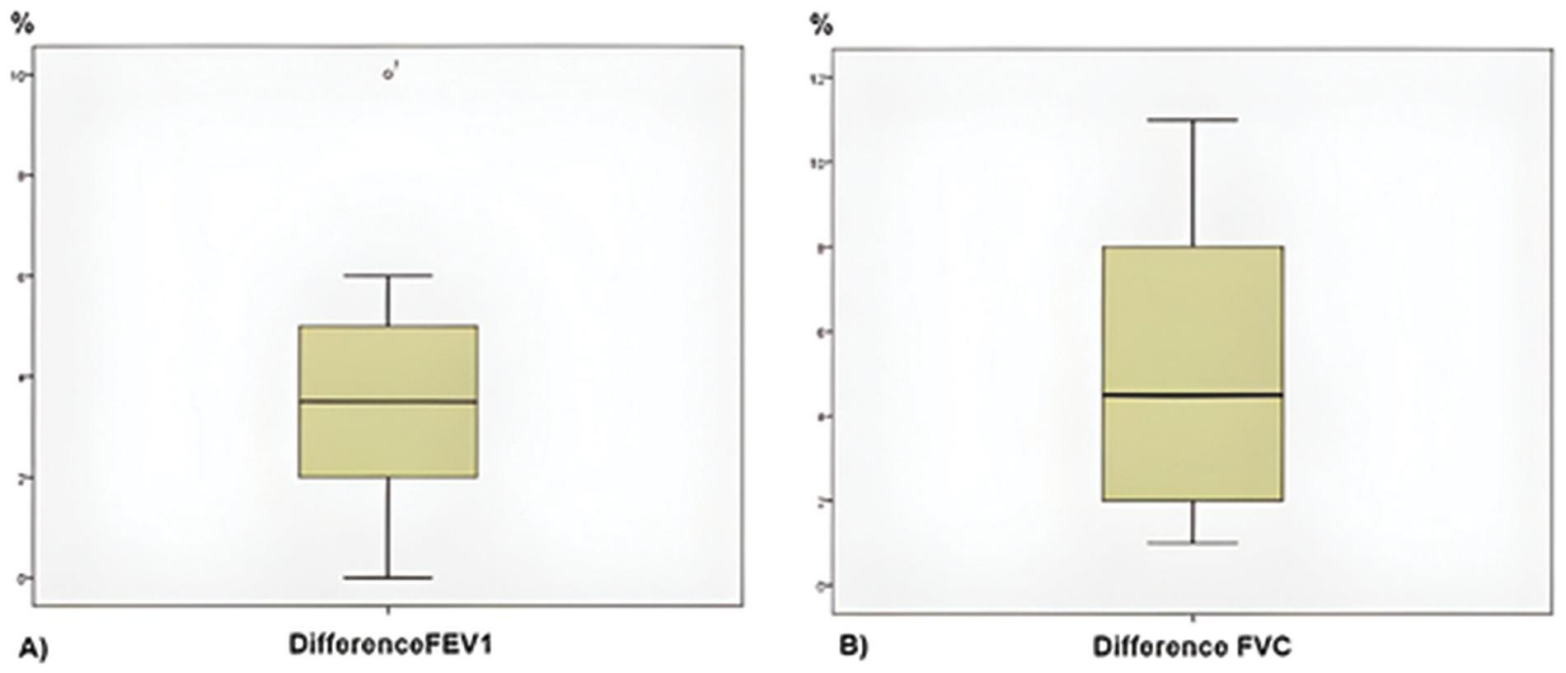
Differences between before and after in FEV1 and FVC. **(A)** FEV1, before vs after. **(B)** FVC, before vs. after.

After 7 days of intervention, there were significant increases in FEV1 (mean difference = 3.88, 95% CI: 1.29 to 6.46, *p* = 0.017, Figure 5A), FVC (mean difference = 5.13, 95% CI: 2.05 to 8.20, *p* = 0.007, Figure 5B), and the FVC/FEV1 ratio (mean difference = 1.25, 95% CI: 0.28 to 2.22, *p* = 0.011). All significant spirometric changes were confirmed using non-parametric Wilcoxon signed-rank tests (Table 3).

**Table 3:** Spirometric variables (% predicted).

Participant	FVC (Pre)	FVC (Post)	FEV₁ (Pre)	FEV₁ (Post)	FEV₁/FVC (Pre)	FEV₁/FVC (Post)
1	67	78	86	96	89	93
2	72	78	87	93	115	116
3	68	75	86	89	101	102
4	76	79	88	89	109	110
5	65	74	80	84	75	76
6	76	78	89	89	94	95
7	78	79	84	88	86	87
8	48	50	66	69	71	71

FVC, Forced vital capacity; FEV1, Forced Expiratory Volume in the first second.

## 5 Discussion

The primary objective of this exploratory study was to describe pulmonary ventilation distribution using electrical impedance tomography (EIT) in women with long COVID and restrictive pulmonary impairment, before and after using the UBICU device. The results showed an increase in intrapulmonary volume and forced vital capacity (FVC) following the intervention period. These preliminary findings suggest a potential association between UBICU use and pulmonary re-expansion and indicate that this approach may represent an innovative tool within physiotherapy practice. While the results are promising and represent an initial step toward new options for care and follow-up, further research with larger samples and controlled designs is essential to confirm and expand upon these observations. Taken together, these findings provide an initial framework for interpreting both functional and regional ventilatory changes associated with respiratory incentive use in this population.

All participants in this study were women. According to the Colombian National Institute of Health, as of March 2024, 53.45% of confirmed COVID-19 cases in Colombia were female (24). This is consistent with findings by Garcia-Molina et al. (25) who reported that 54% of participants in an 8-week outpatient neurorehabilitation program for long COVID patients were women. Several authors have identified female sex as a risk factor for developing long COVID, with a 22% increased probability of experiencing sequelae. These sequelae have been categorized as psychiatric/emotional (OR = 1.80; 95% CI: 1.35–2.41), ENT-related (OR = 1.42; 95% CI: 1.39–1.46), musculoskeletal (OR = 1.15; 95% CI: 1.14–1.16), and respiratory (OR = 1.09; 95% CI: 1.08–1.11), all significantly more prevalent in women (25). The limited sample size, which included only women, restricts the generalizability of the results. Further research in Colombia with larger and more diverse samples is therefore warranted to better characterize sex-related vulnerability and its broader implications.

In the present study, the mean age of participants was 63 ± 9 years. These findings are consistent with previous reports by Taboada et al. (3) and Alarcón et al. (26), who reported mean ages of 65.9 ± 14.1 and 64 ± 9 years, respectively. Although the methodology and focus of those studies differ from ours, the similarity in age-related findings is noteworthy. Despite contextual differences, the alignment of our results with international literature suggests that age ≥60 years may be a relevant factor in the persistence of post–COVID-19 sequelae, underscoring the need for further investigation in local populations.

Although this exploratory study, with a sample of only eight participants, cannot be directly compared with the systematic review and meta-analysis by Torres et al. (27), the findings remain informative. While that review reported a 15% prevalence of restrictive patterns in post–COVID-19 patients, all participants in the present study exhibited restrictive pulmonary impairment. This high prevalence may be related to pulmonary fibrosis (28)and post-infectious scarring, which limit lung expansion. Notably, restrictive impairment showed improvement after seven days of UBICU use, in line with findings reported by Arzayus et al. (4), who described improvements in FVC and FEV₁ following respiratory rehabilitation in post–COVID-19 patients.

It should be noted that plethysmography, the gold standard for confirming restrictive impairment, was not performed due to limited availability in our setting. However, patients with evidence of airflow obstruction were excluded, minimizing the likelihood that the observed reduction in FVC was attributable to obstructive mechanisms. Therefore, the observed changes in FVC and intrapulmonary volume should be interpreted as potentially related to restrictive physiology and the intervention applied.

Previous studies have evaluated incentive spirometry in post–COVID-19 populations. Srinivasan et al. (29) reported improvements in FVC and FEV₁ in 48 post–COVID-19 outpatients using a flow-based incentive spirometer with a prescription of 10 repetitions three times daily for six weeks. These findings are comparable to those observed in the present study, which applied a similar prescription protocol in an outpatient setting and demonstrated an increase in FVC. This effect may be related to increased transpulmonary pressure generated during incentive use, facilitating recruitment of partially collapsed lung regions and increasing intrapulmonary volume. However, spirometric measurements alone do not provide information on how these volumetric changes are spatially distributed within the lung, limiting their mechanistic interpretability.

To address this limitation, pulmonary ventilation distribution was analyzed using EIT before and after UBICU use. EIT is a non-invasive, radiation-free imaging technique originally applied in intensive care settings (30) and later reported in post–COVID-19 patients (31). Scaramuzzo et al. (32) demonstrated persistent regional ventilation inhomogeneity in COVID-19 pneumonia survivors despite normal pulmonary function tests, highlighting that global functional recovery does not necessarily reflect normalization of regional ventilation. In this context, the incorporation of EIT in the present study provides added mechanistic value by enabling assessment of regional ventilation behavior rather than relying solely on global functional indices.

Prior to UBICU use, participants exhibited heterogeneous ventilation with a predominantly dorsal distribution and reduced ventral contribution, particularly in the anterior left region. After seven days of intervention, ventilation increased across all regions of interest, with more consistent gains observed in posterior regions (ROIs 3 and 4) and in the anterior right region (ROI 1). Although dorsal regions remained dominant, ventral regions exhibited proportionally greater increases, leading to a partial reduction in dorsoventral ventilation asymmetry. The limited change observed in ROI 2 may be explained by thoracic anatomy and the position of the heart and great vessels, which mechanically restrict expansion of the left anterior lung (33). These observations suggest that changes in FVC may be associated not only with global lung expansion but also with redistribution of regional ventilation and recruitment of previously under-ventilated areas.

Although the increase in global tidal variation was not statistically significant, the observed trend suggests a possible improvement in intrapulmonary volume through sustained inspiratory efforts that increase lung volume and transpulmonary pressure. The visual feedback and gamification features of UBICU may have contributed to this response by enhancing patient engagement and promoting consistent execution of deep inspiratory maneuvers. The use of EIT further supports the interpretation of these findings by providing objective regional data that complement conventional functional assessments.

The observed changes in pulmonary function and ventilation distribution, together with UBICU's remote monitoring capability, suggest that this device may represent a useful tool for personalized intervention and follow-up in ambulatory rehabilitation settings. This combination of functional assessment, regional mechanistic insight, and remote monitoring is particularly relevant for long COVID patients with persistent ventilatory compromise.

Despite its limitations, this study contributes to the understanding of ventilation distribution and the potential role of incentive spirometry in Colombian women with long COVID. Limitations inherent to EIT include its restriction to a single axial thoracic slice and the use of relative rather than absolute impedance values (34). Additional limitations include the absence of plethysmography, the short intervention duration, and the lack of long-term follow-up, which preclude causal inference and assessment of sustained effects. Nevertheless, the exploratory nature of this study provides a foundation for future research aimed at elucidating the physiological mechanisms of respiratory incentive devices and optimizing rehabilitation strategies in post–COVID-19 populations.

Future studies should incorporate larger samples, longer follow-up periods, and advanced physiological monitoring techniques such as esophageal pressure measurement to better characterize transpulmonary pressure dynamics during incentive use. Randomized controlled trials comparing UBICU with conventional interventions and including standardized lung volume measurements are warranted to validate and expand upon these preliminary findings.

## 6 Conclusions

In eight women with long COVID and restrictive pulmonary impairment, a statistically significant increase in regional tidal variation (TVM) (*p* = 0.007) was observed after UBICU use. These preliminary findings may serve as a basis for future research aimed at evaluating respiratory recovery strategies in outpatient settings, particularly among women with pulmonary sequelae associated with long COVID.

## Data Availability

The raw data supporting the conclusions of this article will be made available by the authors, without undue reservation.

## References

[1] World Health Organization. Post COVID-19 condition (Long COVID) (2022). https://www.who. int/europe/news-room/fact-sheets/item/post-covid-19-condition (Accessed February 25, 2026).

[2] Radovanovic D D’Angelo E. Lung pathophysiology in patients with long COVID-19: one size definitely does not fit all. ERJ Open Res. (2023) 9(2):00052–2023. 10.1183/23120541.00052-2023 37077548 PMC10107065

[3] Taboada M Cariñena A Moreno E Rodríguez N Domínguez MJ Casal A Post-COVID-19 functional status six-months after hospitalization. J Infect. (2021) 82(4):e31–3. 10.1016/j.jinf.2020.12.022 33373650 PMC7834022

[4] Arzayus-Patiño L Perez-Hortua V Aguilar-Zambrano J Asencio-Santofimio H Wilches-Luna EC. Effectiveness of incentive spirometry on lung function in adult COVID- 19 in the acute and post-COVID-19 phase: exploratory review. Curr Respir Med Rev. (2023) 19(3):218–27. 10.2174/1573398X19666230510142030

[5] Carrillo-Esper R. Síndrome pos-COVID-19. Gac Médica México. (2022) 158(3):121–3. 10.24875/gmm.22000072 35894756

[6] Torres-Cantero AM Álvarez León EE Morán-Sánchez I San Lázaro Campillo I Bernal Morell E Hernández Pereña M El impacto de la pandemia de COVID-19 sobre la salud. Informe SESPAS 2022. Gac Sanit. (2022) 36:S4–12. 10.1016/j.gaceta.2022.02.008 35781147 PMC9244867

[7] Lopez-Leon S Wegman-Ostrosky T Perelman C Sepulveda R Rebolledo PA Cuapio A More than 50 long-term effects of COVID-19: a systematic review and meta-analysis. Sci Rep. (2021) 11(1):1. 10.1038/s41598-021-95565-8 34373540 PMC8352980

[8] O’Mahoney LL Routen A Gillies C Ekezie W Welford A Zhang A The prevalence and long-term health effects of long COVID among hospitalised and non-hospitalised populations: a systematic review and meta-analysis. eClinicalMedicine. (2023) 55:101762. 10.1016/j.eclinm.2022.101762 36474804 PMC9714474

[9] Bohmwald K Diethelm-Varela B Rodríguez-Guilarte L Rivera T Riedel CA González PA Pathophysiological, immunological, and inflammatory features of long COVID. Front Immunol. (2024) 15:1341600. 10.3389/fimmu.2024.1341600 38482000 PMC10932978

[10] Fernández-de-las-Peñas C Martín-Guerrero JD Pellicer-Valero ÓJ Navarro-Pardo E Gómez-Mayordomo V Cuadrado ML Female sex is a risk factor associated with long-term post-COVID related-symptoms but not with COVID-19 symptoms: the LONG-COVID-EXP-CM multicenter study. J Clin Med. (2022) 11(2):413. 10.3390/jcm11020413 35054108 PMC8778106

[11] Bai F Tomasoni D Falcinella C Barbanotti D Castoldi R Mulè G Female gender is associated with long COVID syndrome: a prospective cohort study. Clin Microbiol Infect Off Publ Eur Soc Clin Microbiol Infect Dis. (2022) 28(4):611.e9–611.e16. 10.1016/j.cmi.2021.11.002 PMC8575536 34763058

[12] Bwire GM. Coronavirus: why men are more vulnerable to COVID-19 than women? Sn Compr Clin Med. (2020) 2(7):874–6. 10.1007/s42399-020-00341-w 32838138 PMC7271824

[13] Dimas C Alimisis V Uzunoglu N Sotiriadis PP. Advances in electrical impedance tomography inverse problem solution methods: from traditional regularization to deep learning. IEEE Access. (2024) 12:47797–829. 10.1109/ACCESS.2024.3382939

[14] Safaee Fakhr B Araujo Morais CC De Santis Santiago RR Di Fenza R Gibson LE Restrepo PA Bedside monitoring of lung perfusion by electrical impedance tomography in the time of COVID-19. BJA Br J Anaesth. (2020) 125(5):e434–6. 10.1016/j.bja.2020.08.001 32859359 PMC7413127

[15] admin. Ultrasonography and electrical impedance as assessment tools for critical patients in times of COVID-19. Revista Chilena de Anestesia. 2021 September 21. Available online at: https://revistachilenadeanestesia.cl/revchilanestv5007061412/ (Accessed 2025 April 13)

[16] Jonkman AH Alcala GC Pavlovsky B Roca O Spadaro S Scaramuzzo G Lung recruitment assessed by electrical impedance tomography (RECRUIT): a multicenter study of COVID-19 acute respiratory distress syndrome. Am J Respir Crit Care Med. (2023) 208(1):25–38. 10.1164/rccm.202212-2300OC 37097986 PMC10870845

[17] Westwood K Griffin M Roberts K Williams M Yoong K Digger T. Incentive spirometry decreases respiratory complications following major abdominal surgery. Surg J R Coll Surg Edinb Irel. (2007) 5(6):339–42. 10.1016/s1479-666x(07)80086-2 18080608

[18] Campos NE Heinzmann-Filho JP Becker NA Schiwe D Gheller MF de Almeida IS Evaluation of the exercise intensity generated by active video gaming in patients with cystic fibrosis and healthy individuals. J Cyst Fibros Off J Eur Cyst Fibros Soc. (2020) 19(3):434–41. 10.1016/j.jcf.2020.01.001 31928975

[19] Chen YH Hsieh YS. A narrative review of impact of incentive spirometer respiratory training in long COVID. Int J Gen Med. (2024) 17:5233–46. 10.2147/IJGM.S492772 39559556 PMC11570525

[20] Spruit MA Singh SJ Garvey C ZuWallack R Nici L Rochester C An official American thoracic society/European respiratory society statement: key concepts and advances in pulmonary rehabilitation. Am J Respir Crit Care Med. (2013) 188(8):e13–64. 10.1164/rccm.201309-1634ST 24127811

[21] Aguilar-Zambrano J Wilches-Luna EC Asencio-Santofimio H Valencia M Riveros D Navarro A UBICU: a gamified respiratory incentive system for pulmonary Re-expansion. IEEE Access. (2025) 13:15244–52. 10.1109/ACCESS.2025.3528754

[22] Betancourt Peña J Ávila Valencia JC Palacios Gómez M Rodríguez Castro J Benavides Córdoba V. Traducción y adaptación cultural de la escala the post-COVID-19 functional Status (PCFS) scale al español (Colombia). Rev Cuba Investig Bioméd. (2021) 40(Extra 1):18.

[23] Drägerwerk AG & Co. Intrucciones de Uso PulmoVista 500 SW 1.3n [Internet]. (2020). https://www.draeger.com/es_csa/Products/PulmoVista-500 (Accessed March 26, 2026).

[24] Instituto Nacional de Salud. Noticias coronavirus-personal-salud. (2024). https://www.ins.gov.co/ Noticias/Paginas/coronavirus-personal-salud.aspx (Accessed March 18, 2024).

[25] Sylvester SV Rusu R Chan B Bellows M O’Keefe C Nicholson S. Sex differences in sequelae from COVID-19 infection and in long COVID syndrome: a review. Curr Med Res Opin. (2022) 38(8):1391–9. 10.1080/03007995.2022.2081454 35726132

[26] Alarcón M AM Vidal H A Neira Rozas J. Salud intercultural: elementos para la construcción de sus bases conceptuales. Rev Médica Chile. (2003) 131(9):1061–5. 10.4067/S0034-98872003000900014 14635595

[27] Torres-Castro R Vasconcello-Castillo L Alsina-Restoy X Solis-Navarro L Burgos F Puppo H Respiratory function in patients post-infection by COVID-19: a systematic review and meta-analysis. Pulmonology. (2021) 27(4):328–37. 10.1016/j.pulmoe.2020.10.013 33262076 PMC7687368

[28] das Bridi GP Tanni SE Baldi BG. Current understanding of post-COVID pulmonary fibrosis: where are we? Arch Bronconeumol. (2023) 59(2):69–70. 10.1016/j.arbres.2022.07.014 36041958 PMC9395235

[29] Srinivasan V Kandakurti PK Alagesan J Suganthirababu P Kishore Jebasingh T Jenifer Augustina S Efficacy of pursed lip breathing with bhastrika pranayama vs incentive spirometry in rehabilitating post COVID 19 follow up-a randomized control study. Turk J Physiother Rehabil. (2021) 32(3):402–7.

[30] van der Zee P Somhorst P Endeman H Gommers D. Electrical impedance tomography for positive End-expiratory pressure titration in COVID-19–related acute respiratory distress syndrome. Am J Respir Crit Care Med. (2020) 202(2):280–4. 10.1164/rccm.202003-0816LE 32479112 PMC7365366

[31] Katzer K Gremme Y Moshmosh Alsabbagh M Stallmach A Reuken P Lewejohann JC. Electrical impedance tomography (EIT) in a patient suffering from post-COVID syndrome with dyspnea: a case report. Diagnostics. (2022) 12(10):2284. 10.3390/diagnostics12102284 36291973 PMC9599970

[32] Scaramuzzo G Ronzoni L Campo G Priani P Arena C La Rosa R Long-term dyspnea, regional ventilation distribution and peripheral lung function in COVID-19 survivors: a 1 year follow up study. BMC Pulm Med. (2022) 22:408. 10.1186/s12890-022-02214-5 36352423 PMC9643983

[33] Ericsson E Tesselaar E Sjöberg F. Effect of electrode belt and body positions on regional pulmonary ventilation- and perfusion-related impedance changes measured by electric impedance tomography. PLoS One. (2016) 11(6):e0155913. 10.1371/journal.pone.0155913 27253433 PMC4890811

[34] Sanitarias RAP de E de T. Uso de tomógrafo por impedancia eléctrica en pacientes con COVID-19 (2021). Available online at: https://fi-admin.bvsalud.org/document/view/5d9eg (Accessed 2024 April 28)

